# Monitoring activities of teenagers to comprehend their habits: study protocol for a mixed-methods cohort study

**DOI:** 10.1186/1471-2458-13-649

**Published:** 2013-07-12

**Authors:** Mathieu Bélanger, Isabelle Caissie, Jacinthe Beauchamp, Jennifer O’Loughlin, Catherine Sabiston, Michelina Mancuso

**Affiliations:** 1 Centre de formation médicale du Nouveau-Brunswick, Pavillon J.-Raymond-Frenette, 15, rue des Aboiteaux, Moncton, NB E1A 3E9, Canada; 2 Department of family medicine, Université de Sherbrooke, Sherbrooke, Canada; 3 Research Centre, Vitalité Health Network, Moncton, Canada; 4 Department of social and preventive medicine, Université de Montréal, Montreal, Canada; 5 Centre de recherche du Centre Hospitalier de l’Université de Montréal, Montreal, Canada; 6 Institut national de santé publique du Québec, Montreal, Canada; 7 Faculty of Kinesiology and Physical Education, University of Toronto, Toronto, Canada; 8 New Brunswick Health Council, Moncton, Canada

**Keywords:** Physical activity, Sport, Youth, Behavior, Adolescents, Mixed methods, Cohort

## Abstract

**Background:**

Efforts to increase physical activity in youth need to consider which activities are most likely to be sustained over time in order to promote lifelong participation in physical activity. The Monitoring Activities of Teenagers to Comprehend their Habits (MATCH) study is a prospective cohort study that uses quantitative and qualitative methods to develop new knowledge on the sustainability of specific physical activities.

**Methods/design:**

Eight hundred and forty-three grade 5 and 6 students recruited from 17 elementary schools in New Brunswick, Canada, are followed-up three times per year. At each survey cycle, participants complete self-report questionnaires in their classroom under the supervision of trained data collectors. A sub-sample of 24 physically active students is interviewed annually using a semi-structured interview protocol. Parents (or guardians) complete telephone administered questionnaires every two years, and a health and wellness school audit is completed for each school.

**Discussion:**

MATCH will provide a description of the patterns of participation in specific physical activities in youth, and enable identification of the determinants of maintenance, decline, and uptake of participation in each activity. These data will inform the development of interventions that take into account which activities are the most likely to be maintained and why activities are maintained or dropped.

## Background

In Canada, physical inactivity is the most prevalent preventable risk factor for chronic disease and mortality in youth and across the lifespan [1,2]. A recent objective assessment of physical activity in a representative sample of Canadians aged 6 to 19 years indicated that only 9% of boys and 4% of girls engaged in the recommended 60 minutes of moderate-to-vigorous physical activity per day [3]. The burden of chronic disease will likely increase given that low levels of physical activity in youth is a strong predictor of a sedentary lifestyle in adulthood [4].

Physical inactivity in youth increases the risk of cardiovascular disease [5,6], diabetes [7], low bone mineral density [8,9], slower cognitive development [10-12], anxiety and depression [13], and obesity [14,15]. Physical activity and sports provide opportunities for developing competence, experiencing achievement, developing identities, forming positive relationships, learning to respect others, and enhancing a sense of community, while physical inactivity may result in suboptimal psychological and social growth [3]. Enrolment in physical activity programs is associated with improved social and communication skills, heighted motivation, lower rates of deviant behaviours, and better academic achievement [16].

Declines in physical activity during adolescence are characterized by marked changes in the range of physical activities in which youth engage [17]. The popularity of nearly all types of physical activity declines during adolescence [18] and the number of different activities in which adolescents engage also decreases with age [17,19]. With the possible exceptions of active transportation (i.e., walking, bicycling to and from school) and household chores, few young people maintain involvement in specific types of physical activity during adolescence [20-26].

Other reports indicate that the relative contribution of different types of physical activity changes with age. For example, although participation in both organized and non-organized physical activity decreases during adolescence [27-29], there are steeper declines in organized physical activity especially in girls [30]. Similarly, high drop-out rates from sports are reported from childhood to adolescence [18] and from adolescence to adulthood [22,31,32]. Some activities are engaged in almost exclusively by some age groups [31].Skipping rope, playing tag, and using playgrounds are popular among children [24,25], whereas adults engage in physical conditioning and occupational physical activity [31]. Participation in specific physical activities in adulthood is nevertheless more likely among individuals who engaged in the activity during adolescence [22].

Systematic reviews have identified factors that are robustly associated with physical activity [33-35]. However, other than gender differences whereby boys prefer competitive sports [20,21,36-38] and accumulate more vigorous physical activity than girls (who accumulate more moderate physical activity [20,21] and prefer non-competitive individual sports [37]), little is known about the determinants of participating in specific physical activities. Moreover, in-depth understanding of why adolescents discontinue, maintain or initiate physical activity participation is lacking.

Whereas qualitative studies provide rich descriptive information on how various factors influence behavioral patterns, few qualitative studies explore reasons for participation in physical activity during adolescence. Extant qualitative studies show that common reasons for taking part in physical activity include enjoyment, social interaction and weight management, whereas lacking confidence and ability are barriers [39-42]. A qualitative study that explored differences between physical activity maintainers and decliners [43] suggested that decliners reported negative social interactions, unsupportive social environments and feeling insufficiently competent as factors related to declines in physical activity. In contrast, maintenance of physical activity was associated with recognition of health benefits of physical activity, relatedness to others, and perceiving the environment as supportive of physical activity. Insights on factors explaining differences in patterns of physical activity participation during adolescence from both prospective qualitative and quantitative data are needed.

Monitoring Activities of Teenagers to Comprehend their Habits (MATCH) is a prospective mixed-methods study aimed at identifying and better understanding the determinants of discontinuing or sustaining participation in specific physical activities during adolescence. The objectives are: 1) to better understand the physical activity-related experiences of participants in various types of physical activity, including individual activities, team-based activities, organised activities, and non-organised activities; 2) to develop better understanding of the process of maintaining adequate physical activity levels during the transition from childhood to adolescence; 3) to develop better understanding of the process of declining physical activity levels during the transition from childhood to adolescence; 4) to identify determinants of maintaining, discontinuing, or taking up participation in different types of physical activity from childhood to adolescence; and 5) to assess how changes in correlates relate to changes in participation in different types of physical activity.

## Methods/design

### Design and conceptual framework

MATCH is a prospective cohort study that uses quantitative and qualitative methods to develop new knowledge on the natural course and determinants of physical activity in youth. The MATCH study is grounded in the Self-Determination Theory (SDT) which enables better understanding of what motivates people to engage in, and maintain, certain types of physical activity. This in turn will help inform the design of interventions tailored to individual needs. SDT is founded on the notion that individuals behave according to interactions between extrinsic forces, intrinsic motives and essential needs [44]. An integral component of SDT is the underlying theory of Basic Psychological Needs, which assumes that a person maintains optimal functioning in contexts that support three basic psychological needs: competence, autonomy and relatedness [45,46]. Specifically, maintenance of participation in a physical activity is achieved when an individual feels that he or she masters the activity (competence), when the activity is undertaken by choice (autonomy), and when a meaningful connection is established with people through participation in the activity (relatedness). Chaos Theory is also incorporated in components of MATCH because random external events (i.e., loss of a friend, a public announcement, a conversation) may trigger at least short-term behavior change [47]. Chaos Theory supports investigating the influence of unplanned or uncontrolled factors that lead to sudden changes in physical activity. MATCH received Ethics Approval from the *Comité d’Éthique de la Recherche du Centre Hospitalier de l’Université de Sherbrooke*.

### Study population

Nineteen schools were initially recruited from across the province of New Brunswick, Canada to participate in MATCH. However two schools were subsequently excluded because of a low return rate of consent forms. Schools recruited were selected to include a mix of French and English language schools from high, moderate, and low socioeconomic neighbourhoods, situated in rural and urban areas. Students were recruited within the 17 study schools from September 2011 to January 2012. Information packages were sent to the parents of all children in Grade 5 and 6 (10-12 years old), which provided detailed information on the MATCH study objectives and methods, as well as a consent form for parents and students to complete and return to the school. A total of 802 eligible students were recruited in the first year of data collection, for a response proportion of 51%.

Contact information for parents or guardians of 490 of these 802 participants were obtained from schools. At least three attempts to contact each parent were made between August 2012 and February 2013. A total of 253 parents were contacted, of whom 246 (97%) agreed to respond to a telephone-administered questionnaire.

For the qualitative component of the study, a purposely-selected sample of participants was selected from among those categorized, after four survey cycles, as most physically active (we ascertained that four questionnaires were needed to appropriately assess types of activities usually practiced). Six participants were recruited to represent each of the following categories of physical activity participation: involved primarily in a) team activities; b) individual activities; c) organised activities; and d) non-organised activities, for a total of 12 boys and 12 girls. Although overlap occurs among the types of physical activity practiced by different groups, the decision to recruit based on this categorisation was motivated by the intention to include participants with a wide variety of behavioral patterns and not to obtain four mutually exclusive groups. This way, we maximize our ability to develop an understanding of experiences of participation in different types of physical activities.

### Quantitative data collection

Participants provide data three times each year until the end of grade 12 when they graduate from high school, for a total of up to 24 survey cycles per participant. Three survey cycles per year permit investigation of seasonal variation in activity levels and types of activity practiced [48]. At each survey cycle, participants complete self-report questionnaires in their classroom under the supervision of trained data collectors. Classroom visits in the first survey cycle took approximately 45-60 minutes and subsequent visits take approximately 20-30 minutes (students need less instructions). All data collection visits are scheduled at the convenience of teachers.

Data are collected from parents (or guardians) every two years a telephone-administered questionnaires.

During the first year that schools participate in the study, a designated person within each school completes a questionnaire in consultation with other staff, which collects data on the school environment. Responses are verified on-site by a research assistant.

### Student questionnaire

The student questionnaire uses a checklist of 36 activities to collect data on the types of physical activity in which participants engaged over the past four months. The checklist incorporates all of the commonly practiced activities by youth in this region [49] and all activities included in other similar and validated physical activity checklists [50-52]. Participants report the frequency of participation in each activity, where the activity took place (school, home or neighborhood, indoor arena or gym, outdoor field, other), and with whom (by myself, organized group or team, siblings, friends, parents) activities were engaged in [24]. Pilot-testing of the questionnaire in English and French followed by discussions with 12 Grade 5 and 6 students indicated that children have no difficulty understanding and answering the questions.

Physical activity level is estimated using a simple 2-item questionnaire developed for use among youth [53]. This questionnaire has demonstrated test-retest reliability (*r* = 0.77) and it correlated significantly with accelerometer data (*r* = 0.40; this criterion validity index is as good as or better than other physical activity questionnaires) [53].

Basic Psychological Needs are measured in three questionnaires: (i) perceived competence is assessed with the 6-item subscale from the Intrinsic Motivation Inventory [54], (ii) perceived autonomy is assessed in the seven items from the autonomy subscale of the Basic Psychological Needs in Life Scale [55,56], and (iii) perceived relatedness is assessed with the Relatedness to Others in Physical Activity Scale [57]. Each of these scales has internal consistency reliability coefficients (Cronbach’s alpha) of 0.70 to 0.92 and is associated with physical activity [54-57]. Motives for physical activity is measured with the Motivations for Physical Activities Measure-Revised, a 30-item questionnaire that reflects five general motives for physical activity participation including enjoyment, competence, appearance, fitness and social interaction [58]. Enjoyment and competence are measures of intrinsic motives, whereas appearance, fitness and social are indicators of extrinsic motives [58]. The questionnaire has been used successfully to document a relationship between different types of motives for physical activity and long-term adherence to two specific types of physical activity (Tae Kwon Do and Aerobics) [58].

Active transportation is determined by asking youth how they usually get to and from school (actively, inactively or mixed) [59]. The core questionnaire also collects data on age, sex, school-based physical activities [59], sedentary behaviors [60,61], perceived physical activity of others, weight regulating behaviors [62], sleep patterns [63], pubertal stage [64,65], and unplanned events that may have influenced physical activity participation such as parental divorce, sickness in the family, difficulties at school, etc. Data on dietary habits are collected once a year, using items from the 2010 National Youth Physical Activity and Nutrition survey [66].

### Parent questionnaire

Parents provide data on their own level of participation in physical activity in a telephone-administered questionnaire [67]. They also report on their participation in specific physical activities [67], and they provide data on family structure, socioeconomic level, and neighbourhood attributes [68].

Student and parent questionnaires were developed in English and translated into French if validated translations were not already available. The translation was undertaken by a bilingual kinesiologist whose mother tongue is French, with emphasis on conceptual rather than literal translations, and on clear and concise formulation. Three bilingual research team members reviewed and edited the translation for consistency with the English version. The French items were then back-translated by an independent individual whose mother tongue is English. The initial and final English versions where then compared to confirm consistency.

### School questionnaire

The school questionnaire includes data on facilities available for physical activity inside schools, school yards and in the school neighborhood. Data are also collected on whether students have access to schools facilities during non-instructional times throughout the school day, if activities are offered to them when they remain indoors due to inclement weather during non-instructional time, and if students have access to school facilities outside school hours. We also collect information on frequency and duration of Physical Education classes, and school policies related to physical activity and nutrition (adapted from [69] and [70]). Data collected in this school questionnaire has a high level of validity (% agreement with consensus-based opinion among school staff is perfect for >75 of items), test-retest reliability (exact agreement for 79.4% of items), and inter-judge reliability (77.3% of items have shown exact agreement between raters) [71].

### Qualitative data collection

The annual individual qualitative interviews with a sub-sample of 24 participants take place in a private room provided by schools. A phenomenological approach, which is particularly useful for understanding experiences of individuals and gaining insight into their motivations and actions, is used in the qualitative component of this study [72]. The phenomena under study include participation in specific types of physical activity, maintenance of physical activity, and decline in level of physical activity. Consistent with standard phenomenological methodology, our design involves multiple face-to-face interviews [73,74]. Participants are interviewed individually on a yearly basis to describe the evolution of their physical activity-related experiences as it relates to i) the type of physical activity they take part into as well as ii) maintenance or decline in participation in physical activity. The first interview serves as a baseline from which individual profiles will thereafter be built using data from follow up interviews. The interviews are audio-recorded and transcribed verbatim.

Although phenomenological research often begins from a perspective free from hypotheses or preconceptions [75], more recent viewpoints suggest that it is impossible to begin without preconceptions or bias and therefore emphasize that researchers should state their initial theoretical basis [76,77]. Therefore, the semi-structured interview guide (used as a flexible template rather than a rigid list of questions [78]) aims at gathering data on participants’ general experiences with physical activity and physical activity-related feelings of competence, relatedness with others, and autonomy (Basic Psychological Needs component of the SDT). Interviewers are also trained to examine the occurrence of random external triggers if marked changes in patterns of physical activity are noted.

In addition to the 24 participants above, we will invite participants with low physical activity in the first two years of study who subsequently become physically active for interviews that explore circumstances that led to this (sudden) change. Participants in these interviews will be met only once. These interviews could take place in year 3-6, depending on when sudden increases are noted. This will be of particular use to investigate the extent to which “chaotic events” influence increases in physical activity. Interviews will continue until data saturation is reached. All interviews will be recorded and transcribed.

### Limitations

The study schools for MATCH represent a convenience sample. However, the schools were selected specifically to represent a mix of schools from low, middle, and higher socioeconomic status in a variety of urban, suburban, and rural settings in French and English regions of New Brunswick. Further, it is unlikely that the determinants of the maintenance or discontinuation of physical activity differ markedly between youth populations in Canada. It is possible that repeated data collection using the same questionnaire could sensitize participants to motives for maintaining or not participating in physical activity. This is partly why (in addition to cost, feasibility, and seasonality) we limited data collection to every 4^th^ month, rather than monthly or more frequently. Given the young age of participants, it is also possible that they experience difficulty expressing their feelings during the qualitative interviews. Our team includes researchers experienced in conducing in-depth interviews with this age group and has developed strategies to ease this process.

## Discussion

The MATCH study provides the infrastructure for a research program that will generate better understanding of how physical activity participation evolves during childhood and adolescence. Recruiting grade 5 and 6 students and following them throughout adolescence is motivated by the finding that, for most people, peak physical activity level occurs between grade 5-7 [79], and then declines markedly from grade 8-11 [20,21,80-82]. The MATCH study will provide a detailed assessment of the natural history of physical activity participation in a period characterised by important changes in behaviour, growth, and puberty. No study has yet provided such detailed prospective data. Moreover, although the determinants of youth engaging in specific types of physical activities may vary, few investigations distinguish between types of physical activity. MATCH will enable investigating processes of sustaining, interrupting, or initiating participation in a wide variety of different physical activities. Particularly, although some studies have shown strong relationships between different types of physical activity motives and basic psychological needs and physical activity, MATCH has the capacity to tease out why such factors are important for some people, and if this changes over time and for different types of physical activities. In addition, the mixed methods approach enables investigation according to various theoretical frameworks. We will quantify the importance of several potential determinants of participation and develop in-depth understanding of how these determinants arise and co-occur. This combination of approaches will allow gaining insights into processes and events that lead to behavioural changes while also enabling unexpected questions to occur. All of this will guide the development of better interventions aimed at increasing and sustaining participation in physical activity among youth.

## Abbreviations

SDT: Self-Determination Theory; MATCH: Monitoring Activities of Teenagers to Comprehend their Habits

## Competing interests

The authors declare that they have no competing interests.

## Authors’ contributions

MB conceptualized the study and its design. JB, JOL, MM and CS contributed to the conception of the study and study design. IC and MB organize and coordinate the data collection process. MB and IC drafted the first version of this manuscript. All authors reviewed the manuscript for intellectual content and approved it for publication.

## Pre-publication history

The pre-publication history for this paper can be accessed here:

http://www.biomedcentral.com/1471-2458/13/649/prepub
